# Salt stress decreases seed yield and postpones growth process of canola (*Brassica napus* L.) by changing nitrogen and carbon characters

**DOI:** 10.1038/s41598-022-22815-8

**Published:** 2022-10-25

**Authors:** Long Wang, Qingsong Zuo, Jingdong zheng, Jingjing You, Guang Yang, Suohu Leng

**Affiliations:** 1 Jiangsu Key Laboratory of Crop Genetics and Physiology, Agricultural College of Yangzhou University, Yangzhou University, 48 Wenhui East Road, Yangzhou, 225009 Jiangsu China; 2 Jiangsu Co-Innovation Center for Modern Production Technology of Grain Crops, Jiangsu Key Laboratory of Crop Cultivation and Physiology, Agricultural College of Yangzhou University, Yangzhou University, 48 Wenhui East Road, Yangzhou, 225009 Jiangsu China

**Keywords:** Salt, Photosynthesis, Leaf development

## Abstract

Salt stress is a major challenge for plant growth and yield achievement in canola (*Brassica napus* L.). Nitrogen (N) is considered as an essential nutrient involved in many physiological processes, and carbon (C) is the most component of plant biomass. N and C assimilations of canola plants are always inhibited by salt stress. However, the knowledge of how salt stress affects biomass and seed yield through changing N and C characters is limited. A field experiment was conducted to investigate the growth process, N and C characters, photosynthetic performance, biomass accumulation and seed yield under the low and high soil salt-ion concentration conditions (LSSC and HSSC). The results indicated that HSSC postponed the time of early flowering stage and maturity stage by 4 ~ 5 days and 6 ~ 8 days, respectively, as compared with LSSC. Besides, HSSC decreased the N and C accumulation and C/N at both growing stages, suggesting that salt stress break the balance between C assimilation and N assimilation, with stronger effect on C assimilation. Although the plant N content under HSSC was increased, the photosynthesis rate at early flowering stage was decreased. The leaf area index at early flowering stage was also reduced. In addition, HSSC decreased N translocation efficiency especially in stem, and N utilization efficiency. These adverse effects of HSSC together resulted in reduced biomass accumulation and seed yield. In conclusion, the high soil salt-ion concentration reduced biomass accumulation and seed yield in canola through changing N and C characters.

## Introduction

Salinization of agricultural land has been a major challenge for sustainable development of agriculture. The entire saline soil area is about 9.5 billion ha, which accounts for 10% of arable land in the world^1^. China is in possession of 1/16 of the globe saline-alkali land that constitutes about 21% of the cultivated land in China^2^. Canola (*Brassica napus* L.) is one of the most widely cultivated oil crops in the world because of its healthy fatty acid composition in oil and high protein content in meal^3,4^. Currently, the planting area of canola in China is about 7 million hectares, and canola oil owns the largest proportion of domestic edible vegetable oils in the market^5^.

The adverse effect of salt stress on reducing growth rate, which results in smaller leaves, shorter stature and sometimes fewer leaves^6,7^, is functionally carried out by its initial and primary effects on osmotic pressure^8^. Under the action of osmosis, the uptake of water by root is reduced, and furthermore, salt stress may cause excessive uptake of ions^9^. The excessive ions in plant may cause ion toxicity and metabolism disturbance, which may interfere plant ion uptake and intracellular ion balance, and subsequently inhibit photosynthesis and growth^10,11^. Seed germination and seedling emergence are sensitive to salt stress^12–14^. It has been reported that there are great differences in seed germination among canola varieties under salt condition^15,16^.

Nitrogen (N) as an essential nutrient is important for crop yield achievement^17^. It is a structural component of amino acids and a constituent of all enzymes and involved in many physiological processes^18^. Salt stress usually inhibits N absorption and accumulation in canola, resulting in decreased plant height, stunted growth process and ultimately reduced yield^19^. Besides, carbon (C) is the largest component of plant biomass and constitutes a stable 50% of plant dry weight^20^**.** C and N assimilation are the two most important physiological processes related to plant growth and productivity^21^. Studies have demonstrated that C assimilation including photosynthetic carbon assimilation and carbohydrate transport and utilization are adversely affected in plant exposed to salt stress^22^. Nevertheless, little attention was paid to the effects of salt stress on balance of C and N assimilation in canola. N physiologically translocation from the vegetative organs to the reproductive organs after anthesis stage is the important resource of seed N accumulation^23,24^. It was reported that the N translocation from vegetative organs accounts for 50% of seed N accumulation^25^. However, little was known about the N translocation in canola under salt stress.

In the past, researches about salt stress were mainly conducted on pot experiment. The salt stress in these experiments was artificial and not fully reflect the physicochemical properties of saline soil in natural environment. And these researches mainly focus on seedling stage, rarely on flowering stage and maturity stage, due to the limitation of experiment condition. Nevertheless, seed yield is the key to agricultural production. Therefore, in this study, to improve the knowledge of the effects of salt stress on the growth of canola, a field experiment was carried out to investigate the effects of salt stress on N accumulation and translocation, C accumulation, biomass and seed yield. We hypothesize that: (a) salt stress could decrease seed yield in canola plants; (b) N and C assimilation would be inhibited and the balance between N and C assimilation would be changed under salt stress; (c) salt stress would inhibit N translocation from vegetative organs to seed.

## Results

### Growth process

The results of (Table 1) indicated that soil salt-ion concentration affect the growth process after sowing. Specially, as the soil salinity concentration increased from LSSC to HSSC, the time of early flowering stage after sowing of Yanyouza 3 and Ningza 1818 was postponed by 4 ~ 5 days during two growing seasons. The time of maturity stage after sowing was postponed by 6 ~ 8 days.

**Table 1:** Days of early flowering stage and maturity stage after sowing.

Experimental year	Soil salt-ion concentration	Cultivar	Early flowering stage	Maturity stage
2019–2020	LSSC	Yanyouza 3	167	231
		Ningza 1818	168	232
	HSSC	Yanyouza 3	171	237
		Ningza 1818	173	239
2020–2021	LSSC	Yanyouza 3	168	233
		Ningza 1818	169	234
	HSSC	Yanyouza 3	172	240
		Ningza 1818	173	242

LSSC and HSSC represent low and high soil salt-ion concentration.

### Seed yield

**Figure 1:**
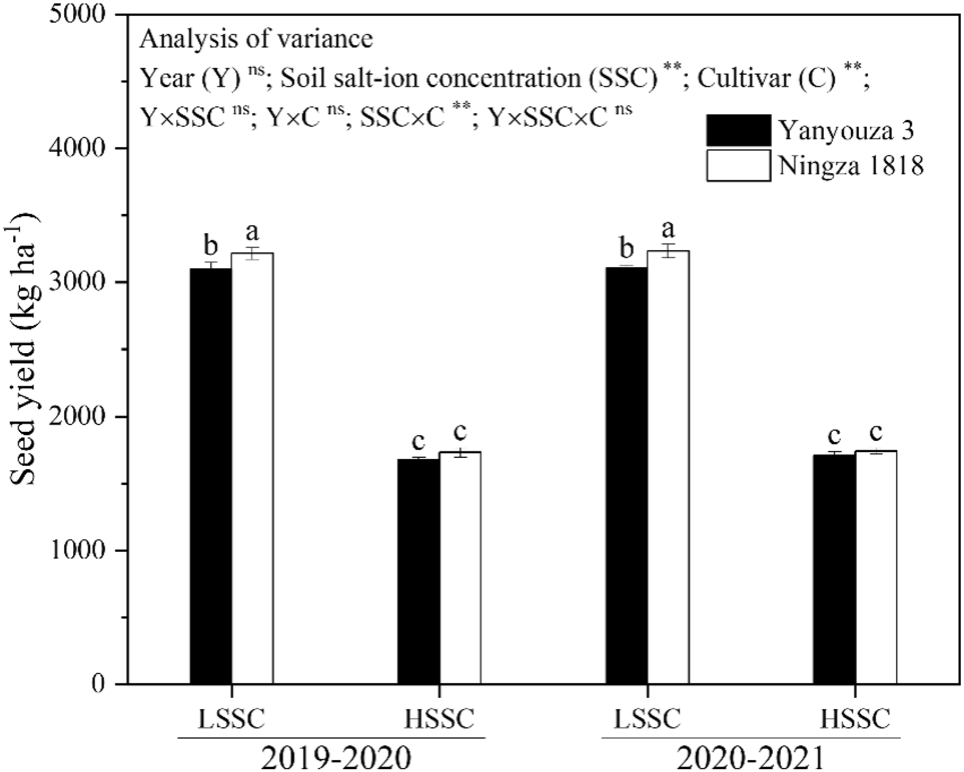
The ANOVA results and seed yield under different treatments. LSSC and HSSC represent low and high soil salt-ion concentration. Probability levels are performed by ns, and ** for not significant, and 0.01. Different letters indicate significant difference at p = 0.05 between different treatments during two growing seasons. Data are mean ± SE (n = 3).

The ANOVA results indicated that soil salt-ion concentration, cultivar and their interaction significantly affected seed yield. The seed yield, ranging from 1679.76 to 3232.34 kg ha^−1^, decreased dramatically with the soil salt-ion concentration increasing from LSSC to HSSC. More specially, the seed yield of Yanyouza 3 under HSSC was reduced by 45.78 and 44.92% during 2019–2020 and 2020–2021 growing seasons, respectively, as compared with that under LSSC. For Ningza 1818, both the reductions of seed yield during two growing seasons were 46.11%. Moreover, Ningza1818 showed greater yield performance than Yanyouza 3 under both LSSC and HSSC conditions (Fig. 1).

### Biomass accumulation

The ANOVA results (Table 2) indicated that soil salt-ion concentration and cultivar significantly affected biomass accumulation in different parts at both early flowering stage and maturity stage, except cultivar effect on root biomass accumulation at both two growth stages; however, year and the interactions showed no significant effect on most of biomass accumulation. The increasing soil salt-ion concentration decreased biomass accumulation in all parts at both growth stages. Averagely over two growing seasons, HSSC treatment significantly decreased biomass accumulation of different pars in Yanyouza 3 and Ningza 1818 by 21.28% and 24.55% (root), 33.03% and 33.92% (stem), 25.64% and 24.99% (leaf), and 36.86% and 34.79% (per-anthesis deciduous leaf) at early flowering stage, as compared with LSSC treatment. These reductions in maturity stage were 26.93% and 26.70% (root), 32.27% and 29.93% (stem), 22.31% and 20.86% (post-anthesis deciduous leaf), 39.25% and 42.64% (pod), and 46.35% and 46.11% (seed), respectively.

**Table 2:** The ANOVA results and biomass accumulation (10^3^ kg ha^−1^) under different soil salt-ion concentration.

Year	Cultivar	Soil salt-ion concentration	Early flowering stage				Maturity stage				
			Root	Stem	Leaf	Pre-anthesis deciduous leaf	Root	Stem	Post-anthesis deciduous leaf	Pod	Seed
2019–2020	Yanyouza 3	LSSC	0.66 ± 0.02a	2.98 ± 0.06b	1.46 ± 0.04c	0.57 ± 0.01a	0.84 ± 0.02a	3.71 ± 0.07b	1.39 ± 0.07b	2.48 ± 0.07c	3.1 ± 0.05b
		HSSC	0.52 ± 0.01b	2.02 ± 0.04c	1.11 ± 0.04e	0.36 ± 0.01c	0.62 ± 0.01b	2.48 ± 0.04d	1.11 ± 0.03d	1.58 ± 0.03d	1.68 ± 0.02c
	Ningza 1818	LSSC	0.66 ± 0.01a	3.12 ± 0.03a	1.58 ± 0.05a	0.57 ± 0.01a	0.86 ± 0.02a	3.86 ± 0.09a	1.5 ± 0.04a	2.73 ± 0.08a	3.21 ± 0.04a
		HSSC	0.52 ± 0.01b	2.04 ± 0.06c	1.19 ± 0.01d	0.37 ± 0.01bc	0.61 ± 0.01b	2.74 ± 0.09c	1.23 ± 0.04c	1.55 ± 0.02d	1.73 ± 0.04c
2020–2021	Yanyouza 3	LSSC	0.68 ± 0.02a	3.05 ± 0.05ab	1.5 ± 0.05bc	0.57 ± 0.01a	0.86 ± 0.03a	3.70 ± 0.07b	1.49 ± 0.05a	2.61 ± 0.04b	3.1 ± 0.02b
		HSSC	0.53 ± 0.01b	2.01 ± 0.03c	1.10 ± 0.01e	0.36 ± 0.01c	0.62 ± 0.02b	2.54 ± 0.11d	1.12 ± 0.03d	1.5 ± 0.02d	1.71 ± 0.03c
	Ningza 1818	LSSC	0.67 ± 0.01a	3.08 ± 0.03a	1.55 ± 0.03ab	0.58 ± 0.02a	0.84 ± 0.01a	3.93 ± 0.09a	1.51 ± 0.05a	2.66 ± 0.04ab	3.23 ± 0.05a
		HSSC	0.52 ± 0.01b	2.05 ± 0.04c	1.16 ± 0.01de	0.38 ± 0.01b	0.64 ± 0.01b	2.72 ± 0.06c	1.16 ± 0.01cd	1.54 ± 0.03d	1.74 ± 0.02c
**ANOVA**											
Soil salt-ion concentration (SSC)			**	**	**	**	**	**	**	**	**
Cultivar (C)			ns	**	**	**	ns	**	**	**	**
Year (Y)			*	ns	ns	ns	ns	ns	ns	ns	ns
SSC*C			ns	ns	ns	ns	ns	ns	ns	**	*
SSC*Y			ns	ns	ns	ns	ns	ns	*	ns	ns
C*Y			ns	ns	ns	ns	ns	ns	*	ns	ns
SSC*C*Y			ns	ns	ns	ns	ns	ns	ns	**	ns

LSSC and HSSC represent low soil salt-ion concentration and high soil salt-ion concentration.

Probability levels are performed by ns, * and ** for not significant, 0.05 and 0.01. Different letters indicate significant difference at p = 0.05 between different treatments during two growing seasons. Data is mean ± SE (n = 3).

### N characters

The ANOVA results (Table 3) showed that soil salt-ion concentration significantly affected N content of all parts at both two growth stages; cultivar significantly affected N content of most parts (except root) at maturity stage and stem N content at early flowering stage. Year and the interactions between two factors or three factors showed no significant effect mostly. As the soil salt-ion concentration increased from LSSC to HSSC, the N content of all parts in Yanyouza 3 and Ningza 1818 was significantly increased at both early flowering stage and maturity stage. For example, as compared with LSSC, HSSC averagely increased the N content of root, stem and leaf in Yanyouza 3 and Ningza 1818 over two growing seasons by 11.86% and 10.95%, 11.47% and 16.25%, 5.29% and 4.52% at early flowering stage. Similarly, HSSC treatment increased N content of root, stem, pod and seed by 21.01% and 21.70%, 33.22% and 29.79%, 18.40% and 14.91%, 8.00% and 9.09% at maturity stage.

**Table 3:** The ANOVA results and N content (%) under different treatments.

Year	Cultivar	Soil salt-ion concentration	Early flowering stage				Maturity stage				
			Root	Stem	Leaf	Pre-anthesis deciduous leaf	Root	Stem	Post-anthesis deciduous leaf	Pod	Seed
2019–2020	Yanyouza 3	LSSC	1.19 ± 0.02c	1.72 ± 0.03d	3.93 ± 0.07 cd	0.86 ± 0.02b	0.65 ± 0.03bc	0.64 ± 0.02c	0.96 ± 0.03 cd	0.74 ± 0.02c	3.69 ± 0.01d
		HSSC	1.30 ± 0.01ab	1.90 ± 0.01b	4.13 ± 0.08a	0.93 ± 0.02a	0.78 ± 0.01a	0.85 ± 0.02a	1.10 ± 0.02ab	0.86 ± 0.02a	3.99 ± 0.08ab
	Ningza 1818	LSSC	1.18 ± 0.01c	1.57 ± 0.03e	3.90 ± 0.07d	0.84 ± 0.02b	0.63 ± 0.01c	0.63 ± 0.02c	0.91 ± 0.02e	0.71 ± 0.03c	3.55 ± 0.08e
		HSSC	1.30 ± 0.02b	1.8 ± 0.03c	4.06 ± 0.07abc	0.93 ± 0.02a	0.78 ± 0.01a	0.80 ± 0.01b	1.09 ± 0.02b	0.80 ± 0.02b	3.84 ± 0.06c
2020–2021	Yanyouza 3	LSSC	1.14 ± 0.01d	1.73 ± 0.01d	3.97 ± 0.13bcd	0.84 ± 0.02b	0.66 ± 0.02b	0.63 ± 0.01c	0.97 ± 0.02c	0.72 ± 0.02c	3.71 ± 0.05d
		HSSC	1.30 ± 0.01ab	1.96 ± 0.03a	4.20 ± 0.08a	0.91 ± 0.02a	0.80 ± 0.01a	0.85 ± 0.01a	1.14 ± 0.01ab	0.87 ± 0.03a	4.00 ± 0.08a
	Ningza 1818	LSSC	1.18 ± 0.03c	1.57 ± 0.03e	3.91 ± 0.07d	0.86 ± 0.02b	0.65 ± 0.01bc	0.62 ± 0.02c	0.94 ± 0.01de	0.70 ± 0.01c	3.53 ± 0.08e
		HSSC	1.33 ± 0.02a	1.84 ± 0.05c	4.1 ± 0.04ab	0.93 ± 0.03a	0.78 ± 0.01a	0.81 ± 0.01b	1.12 ± 0.03ab	0.82 ± 0.02b	3.88 ± 0.06bc
**ANOVA**											
Soil salt-ion concentration (SSC)			**	**	**	**	**	**	**	**	**
Cultivar (C)			ns	**	ns	ns	ns	**	**	**	**
Year (Y)			ns	*	ns	ns	ns	ns	**	ns	ns
SSC*C			ns	*	ns	ns	ns	ns	ns	ns	ns
SSC*Y			*	ns	ns	ns	ns	ns	ns	ns	ns
C*Y			*	ns	ns	ns	ns	ns	ns	ns	ns
SSC*C*Y			ns	ns	ns	ns	ns	ns	ns	ns	ns

LSSC and HSSC represent low soil salt-ion concentration and high soil salt-ion concentration.

Probability levels are performed by ns, * and ** for not significant, 0.05 and 0.01. Different letters within the same column indicate significant difference at p = 0.05 between different treatments during two growing seasons. Data is mean ± SE (n = 3).

However, the effect of soil salt-ion concentration on N accumulation amount was different from that on N content (Table 4). As compared with LSSC, HSSC significantly reduced the N accumulation amount of different parts. The HSSC treatment averagely decreased N accumulation amount of root, stem, leaf and pre-anthesis deciduous leaf in Yanyouza 3 and Ningza 1818 over two growing seasons by 11.94% and 12.97, 25.36 and 23.18%, 21.73% and 21.58%, 31.56% and 28.85% at early flowering stage. These reductions at maturity stage were 11.58% and 10.83% for root, 9.76% and 9.03% for stem, 10.10% and 5.04% for post-anthesis deciduous leaf, 28.08% and 34.08% for pod, 40.99% and 41.21% for seed, respectively. The ANOVA results indicated that soil salt-ion concentration significantly affected N accumulation of all parts, cultivar affected N accumulation at early flowering stage (except root) and stem N accumulation at maturity stage. Year and the interaction between two factors or three factors showed no significant effect mostly.

**Table 4:** The ANOVA results and N accumulation (kg ha^−1^) under different treatments.

Year	Cultivar	Soil salt-ion concentration	Early flowering stage				Maturity stage				
			Root	Stem	Leaf	Pre-anthesis deciduous leaf	Root	Stem	Post-anthesis deciduous leaf	Pod	Seed
2019–2020	Yanyouza 3	LSSC	7.83 ± 0.26a	51.39 ± 1.47a	57.41 ± 2.34b	4.89 ± 0.17a	5.50 ± 0.34a	23.87 ± 0.75a	13.36 ± 0.36 cd	18.30 ± 0.32b	114.26 ± 1.97a
		HSSC	6.76 ± 0.13b	38.44 ± 0.93 cd	45.66 ± 1.07c	3.36 ± 0.06c	4.88 ± 0.09b	20.96 ± 0.23c	12.23 ± 0.25e	13.70 ± 0.52c	66.97 ± 0.71b
	Ningza 1818	LSSC	7.79 ± 0.03a	48.97 ± 1.25b	61.52 ± 1.66a	4.84 ± 0.14a	5.45 ± 0.11a	24.21 ± 0.96a	13.63 ± 0.49bc	19.28 ± 0.89a	114.03 ± 3.91a
		HSSC	6.75 ± 0.20b	36.82 ± 1.68d	48.33 ± 1.02c	3.43 ± 0.07bc	4.78 ± 0.13b	21.98 ± 1.10bc	13.34 ± 0.57 cd	12.39 ± 0.20d	66.45 ± 1.03b
2020–2021	Yanyouza 3	LSSC	7.75 ± 0.20a	52.77 ± 0.58a	59.71 ± 2.92ab	4.82 ± 0.05a	5.65 ± 0.10a	23.27 ± 0.78ab	14.47 ± 0.28a	18.88 ± 0.31ab	115.25 ± 2.37a
		HSSC	6.96 ± 0.06b	39.31 ± 0.89c	45.99 ± 1.12c	3.28 ± 0.12c	4.98 ± 0.17b	21.56 ± 1.16c	12.78 ± 0.44de	13.03 ± 0.56 cd	68.47 ± 0.71b
	Ningza 1818	LSSC	7.87 ± 0.18a	48.21 ± 0.41b	60.60 ± 1.54a	5.00 ± 0.14a	5.49 ± 0.02a	24.33 ± 0.23a	14.16 ± 0.52ab	18.69 ± 0.17ab	113.94 ± 1.26a
		HSSC	6.87 ± 0.08b	37.81 ± 0.48 cd	47.44 ± 1.00c	3.57 ± 0.02b	4.98 ± 0.06b	22.18 ± 0.77bc	13.03 ± 0.28 cd	12.63 ± 0.28d	67.58 ± 1.97b
**ANOVA**											
Soil salt-ion concentration (SSC)			**	**	**	**	**	**	**	**	**
Cultivar (C)			ns	**	**	*	ns	*	ns	ns	ns
Year (Y)			ns	ns	ns	ns	ns	ns	*	ns	ns
SSC*C			ns	*	ns	ns	ns	ns	ns	**	ns
SSC*Y			ns	ns	ns	ns	ns	ns	ns	ns	ns
C*Y			ns	ns	ns	*	ns	ns	*	ns	ns
SSC*C*Y			ns	ns	ns	ns	ns	ns	ns	*	ns

LSSC and HSSC represent low soil salt-ion concentration and high soil salt-ion concentration.

Probability levels are performed by ns, * and ** for not significant, 0.05 and 0.01. Different letters within the same column indicate significant difference at p = 0.05. Data is mean ± SE (n = 3).

The ANOVA results (Table 5) showed that soil salt-ion concentration significantly affected N translocation efficiency of stem and leaf and N utilization efficiency; cultivar significantly affected N translocation efficiency of stem and N utilization efficiency; year and interactions had no significant effect. Similarly, the effects of soil salt-ion concentration on N translocation efficiency followed the same change tendency as N accumulation amount. As compared with LSSC, HSSC averagely decreased the N translocation efficiency of stem and leaf in Yanyouza 3 and Ningza 1818 over two growing seasons by 17.11% and 18.38%, 4.61% and 6.17%, respectively. The HSSC treatment also decreased the N utilization efficiency by 19.03% in Yanyouza 3 and 20.58% in Ningza 1818, as compared with LSSC treatment. Moreover, the correlation analysis indicated that seed yield and seed N accumulation were significantly and positively related with stem and leaf N translocation efficiency.

**Table 5:** The ANOVA and correlation analysis results and N translocation efficiency (%) and N utilization efficiency under different treatments.

Year	Cultivar	Soil salt-ion concentration	N translocation efficiency (%)			N utilization efficiency (kg kg^−1^)
			Root	Stem	Leaf	
2019–2020	Yanyouza 3	LSSC	29.88 ± 2.61ab	53.51 ± 2.81a	76.69 ± 1.47a	17.19 ± 0.18b
		HSSC	27.88 ± 0.39ab	45.44 ± 1.61c	73.21 ± 0.97b	13.76 ± 0.23c
	Ningza 1818	LSSC	29.98 ± 1.35ab	50.57 ± 1.03b	77.85 ± 0.77a	17.72 ± 0.30a
		HSSC	29.27 ± 0.58ab	40.33 ± 0.75d	72.39 ± 0.98b	14.16 ± 0.32c
2020–2021	Yanyouza 3	LSSC	27.04 ± 1.93b	55.90 ± 1.88a	75.74 ± 0.70a	17.03 ± 0.21b
		HSSC	28.45 ± 2.11ab	45.19 ± 1.71c	72.2 ± 1.42b	13.78 ± 0.26c
	Ningza 1818	LSSC	30.29 ± 1.46a	49.53 ± 0.90b	76.61 ± 1.34a	17.80 ± 0.30a
		HSSC	27.58 ± 0.33ab	41.36 ± 1.65d	72.52 ± 1.15b	14.05 ± 0.13c
**ANOVA**						
Soil salt-ion concentration (SSC)			ns	**	**	**
Cultivar (C)			ns	**	ns	**
Year (Y)			ns	ns	ns	ns
SSC*C			ns	ns	ns	ns
SSC*Y			ns	ns	ns	ns
C*Y			ns	ns	ns	ns
SSC*C*Y			ns	ns	ns	ns
**Correlation analysis**						
Seed N accumulation			0.294 ns	0.873**	0.884**	
Seed yield			0.317 ns	0.836**	0.885**	

LSSC and HSSC represent low soil salt-ion concentration and high soil salt-ion concentration.

Probability levels are performed by ns and ** for not significant and 0.01. Different letters within the same column indicate significant difference at p = 0.05 between different treatments during two growing seasons. Data is mean ± SE (n = 3).

### C characters and C/N

The ANOVA results (Table 6) showed that soil salt-ion concentration significantly affected C content in all organs at two growth stages. The range of C content in root, stem, leaf and pre-anthesis deciduous leaf at earling flowering stage were 40.16–41.52%, 38.16–39.83%, 40.18–41.75% and 35.23–37.12%, respectively. At maturity stage, the range of C content in root, stem, post-anthesis deciduous leaf, pod and seed were 40.12–41.32%, 40.43–41.38%, 34.03–35.88%, 38.65–41.09% and 57.06–58.32% respectively. For the one cultivar during one growing seaon, the C content in specific organ under HSSC treatment mostly had no significant difference from that under LSSC treatment. However, the C content under HSSC treatment showed mild decline than those under LSSC treatment.

**Table 6:** The ANOVA results and C content (%) under different treatments.

Year	Cultivar	Soil salt-ion concentration	Early flowering stage				Maturity stage				
			Root	Stem	Leaf	Pre-anthesis deciduous leaf	Root	Stem	Post-anthesis deciduous leaf	Pod	Seed
2019–2020	Yanyouza 3	LSSC	41.43 ± 0.38a	39.75 ± 0.68a	41.47 ± 0.77ab	36.42 ± 0.06abc	41.32 ± 0.28a	40.96 ± 0.35a	35.22 ± 0.84ab	40.82 ± 0.54ab	58.16 ± 0.45ab
		HSSC	40.16 ± 0.57c	38.44 ± 0.30ab	40.18 ± 0.60b	35.64 ± 0.24cde	40.52 ± 0.44ab	40.53 ± 0.53a	34.75 ± 1.28ab	40.05 ± 0.45b	57.24 ± 0.41ab
	Ningza 1818	LSSC	41.05 ± 0.32abc	39.24 ± 0.34ab	41.25 ± 0.95ab	36.12 ± 0.18bcd	41.24 ± 0.77a	41.21 ± 0.48a	35.86 ± 0.77a	40.75 ± 0.34ab	58.32 ± 0.61a
		HSSC	40.93 ± 0.41abc	38.16 ± 0.80b	40.72 ± 0.27ab	35.23 ± 0.19e	40.84 ± 0.39ab	40.46 ± 0.46a	34.18 ± 0.52b	38.65 ± 0.60c	57.41 ± 0.53ab
2020–2021	Yanyouza 3	LSSC	41.52 ± 0.47a	39.83 ± 0.96a	41.75 ± 1.05a	37.12 ± 0.81a	41.03 ± 0.59ab	41.02 ± 0.96a	35.88 ± 0.48a	41.01 ± 0.39ab	58.22 ± 0.78ab
		HSSC	40.58 ± 0.58abc	38.69 ± 1.09ab	40.59 ± 0.11ab	35.89 ± 0.24cde	40.44 ± 0.33ab	40.51 ± 0.68a	34.72 ± 0.74ab	40.34 ± 0.76ab	57.06 ± 0.47b
	Ningza 1818	LSSC	41.22 ± 0.77ab	39.52 ± 1.03ab	41.52 ± 0.87ab	36.92 ± 0.45ab	41.01 ± 0.67ab	41.38 ± 0.29a	35.12 ± 0.63ab	41.09 ± 0.29a	58.23 ± 1.03ab
		HSSC	40.28 ± 0.29bc	38.36 ± 0.83ab	40.32 ± 0.80ab	35.41 ± 0.75de	40.12 ± 0.04b	40.43 ± 0.35a	34.03 ± 0.31b	40.12 ± 0.54ab	57.26 ± 0.50ab
**ANOVA**											
Soil salt-ion concentration (SSC)			**	**	**	**	**	**	**	**	**
Cultivar (C)			ns	ns	ns	ns	ns	ns	ns	ns	ns
Year (Y)			ns	ns	ns	*	ns	ns	ns	*	ns
SSC*C			ns	ns	ns	ns	ns	ns	ns	ns	ns
SSC*Y			ns	ns	ns	ns	ns	ns	ns	ns	ns
C*Y			ns	ns	ns	ns	ns	ns	ns	ns	ns
SSC*C*Y			ns	ns	ns	ns	ns	ns	ns	ns	ns

LSSC and HSSC represent low soil salt-ion concentration and high soil salt-ion concentration.

Probability levels are performed by ns, * and ** for not significant, 0.05 and 0.01. Different letters within the same column indicate significant difference at p = 0.05 between different treatments during two growing seasons. Data is mean ± SE (n = 3).

The ANOVA results (Table 7) indicated that soil salt-ion concentration showed significant effect on C accumulation; cultivar significantly mainly affected C accumulation at maturity stage; year and the interactions rarely exerted significant effect on C accumulation. Similar to N accumulation, the increase in soil salt-ion concentration reduced the C accumulation. In contrast to LSSC treatment, the HSSC treatment averagely decreased the C accumulation of root, stem, leaf and pre-anthesis deciduous leaf in Yanyouza 3 and Ningza 1818 at early flowering stage by 23.37% and 22.55%, 35.08% and 35.81%, 27.81% and 26.53%, and 38.58% and 36.94%, respectively. At maturity stage, the reduction of root, stem, post-anthesis deciduous leaf, pod and seed in Yanyouza 3 and Ningza 1818 were 28.16% and 27.85%, 33.03% and 31.37%, 24.04% and 23.96%, 40.32% and 44.78%, and 46.33% and 46.98%, respectively.

**Table 7:** The ANOVA results and C accumulation (kg ha^−1^) under different treatments.

Year	Cultivar	Soil salt-ion concentration	Early flowering stage				Maturity stage				
			Root	Stem	Leaf	Pre-anthesis deciduous leaf	Root	Stem	Post-anthesis deciduous leaf	Pod	Seed
2019–2020	Yanyouza 3	LSSC	273.53 ± 7.80ab	1184.18 ± 23.75b	605.49 ± 8.47c	206.44 ± 2.34b	348.05 ± 7.55a	1519.18 ± 23.32b	490.42 ± 14.31b	1011.94 ± 41.50c	1802.09 ± 42.39b
		HSSC	208.16 ± 5.86c	778.03 ± 17.11c	444.66 ± 11.79f.	128.64 ± 3.25c	252.25 ± 5.27b	1004.78 ± 4.56d	386.14 ± 18.48d	634.75 ± 16.18d	961.52 ± 14.42c
	Ningza 1818	LSSC	269.83 ± 2.93b	1223.89 ± 11.72a	650.14 ± 10.18a	207.38 ± 2.54b	354.97 ± 8.77a	1592.24 ± 53.59a	539.37 ± 9.14a	1111.43 ± 22.66a	1874.52 ± 41.46a
		HSSC	213.43 ± 5.29c	779.40 ± 25.74c	484.27 ± 5.30d	130.52 ± 0.82c	249.73 ± 6.77b	1107.39 ± 36.68c	418.67 ± 11.23c	598.9 ± 0.18d	994.43 ± 24.42c
2020–2021	Yanyouza 3	LSSC	281.21 ± 8.66a	1212.91 ± 13.16ab	626.93 ± 8.83b	213.04 ± 6.05ab	353.70 ± 15.46a	1516.02 ± 32.10b	532.83 ± 10.24a	1068.39 ± 22.83b	1807.77 ± 36.41b
		HSSC	216.98 ± 6.05c	778.05 ± 32.68c	444.78 ± 4.57f.	128.93 ± 1.96c	251.86 ± 10.77b	1027.80 ± 59.63d	389.96 ± 3.63d	605.08 ± 16.41d	975.81 ± 21.40c
	Ningza 1818	LSSC	275.53 ± 2.84ab	1217.03 ± 19.16ab	643.85 ± 12.24a	215.37 ± 8.06a	345.30 ± 3.81a	1625.54 ± 27.62a	531.58 ± 24.46a	1093.34 ± 16.42ab	1882.26 ± 48.19a
		HSSC	208.88 ± 5.39c	787.50 ± 5.85c	466.51 ± 7.68e	136.06 ± 1.60c	255.36 ± 5.14b	1100.60 ± 15.11c	395.76 ± 7.29 cd	618.28 ± 20.1d	997.41 ± 6.67c
**ANOVA**											
Soil salt-ion concentration (SSC)			**	**	**	**	**	**	**	**	**
Cultivar (C)			ns	ns	**	ns	ns	**	**	*	**
Year (Y)			ns	ns	ns	**	ns	ns	ns	ns	ns
SSC*C			ns	ns	ns	ns	ns	ns	ns	**	ns
SSC*Y			ns	ns	*	ns	ns	ns	*	ns	ns
C*Y			ns	ns	**	ns	ns	ns	**	ns	ns
SSC*C*Y			ns	ns	ns	ns	ns	ns	ns	**	ns

LSSC and HSSC represent low soil salt-ion concentration and high soil salt-ion concentration.

Probability levels are performed by ns, * and ** for not significant, 0.05 and 0.01. Different letters within the same column indicate significant difference at p = 0.05 between different treatments during two growing seasons. Data is mean ± SE (n = 3).

The ANOVA results (Table 8) showed that soil salt-ion concentration significantly affected C/N. At early flowering stage, the C/N in pre-anthesis deciduous leaf was the largest (38.06–44.21), followed by root (30.38–36.30) and stem (19.79–25.25), the least one was leaf (9.67–10.63). At maturity stage, the C/N in root, stem and pod were relatively higher (all above 50), followed by post-anthesis deciduous leaf, the least one is seed (14.25–16.52). as the soil salt-ion concentration increasing, the C/N in all organs were significantly deceased. At early flowering stage, the C/N of root, stem, leaf and pre-anthesis deciduous leaf in Yanyouza 3 and Ningza 1818 under HSSC treatment were 12.97% and 11.02%, 13.06% and 16.41%, 7.85% and 6.32%, 10.26% and 11.40% lower than those under LSSC treatment. At maturity stage, the C/N of root, stem, post-anthesis deciduous leaf, pod and seed in Yanyouza 3 and Ningza 1818 under HSSC treatment were 18.83% and 19.11%, 25.79% and 24.54%, 15.50% and 19.88%, 17.04% and 16.27%, 9.03% and 9.81% lower than those under LSSC treatment.

**Table 8:** The ANOVA results and C/N under different treatments.

Year	Cultivar	Soil salt-ion concentration	Early flowering stage				Maturity stage				
			Root	Stem	Leaf	Pre-anthesis deciduous leaf	Root	Stem	Post-anthesis deciduous leaf	Pod	Seed
2019–2020	Yanyouza 3	LSSC	34.92 ± 0.38b	23.05 ± 0.78b	10.56 ± 0.37a	42.27 ± 1.02a	63.45 ± 2.98a	63.68 ± 1.28b	36.70 ± 0.43b	55.29 ± 2.36b	15.77 ± 0.11b
		HSSC	30.78 ± 0.47 cd	20.24 ± 0.08de	9.74 ± 0.06b	38.30 ± 1.08b	51.72 ± 0.28b	47.93 ± 0.73d	31.59 ± 1.57c	46.34 ± 0.59c	14.36 ± 0.37d
	Ningza 1818	LSSC	34.66 ± 0.30b	25.01 ± 0.70a	10.57 ± 0.12a	42.86 ± 0.92a	65.13 ± 1.76a	65.81 ± 2.23ab	39.60 ± 0.90a	57.70 ± 2.45ab	16.44 ± 0.20a
		HSSC	31.60 ± 0.16c	21.17 ± 0.39c	10.02 ± 0.11b	38.06 ± 0.95b	52.28 ± 0.69b	50.42 ± 1.03c	31.39 ± 0.54c	48.34 ± 0.78c	14.96 ± 0.21c
2020–2021	Yanyouza 3	LSSC	36.30 ± 0.75a	22.99 ± 0.47b	10.51 ± 0.36a	44.21 ± 1.4a	62.60 ± 2.37a	65.18 ± 1.54ab	36.82 ± 0.39b	56.60 ± 1.75ab	15.69 ± 0.01b
		HSSC	31.19 ± 0.71 cd	19.79 ± 0.44e	9.67 ± 0.21b	39.29 ± 0.91b	50.59 ± 0.63b	47.68 ± 0.19d	30.54 ± 0.95c	46.47 ± 0.90c	14.25 ± 0.39d
	Ningza 1818	LSSC	35.01 ± 1.15b	25.25 ± 0.38a	10.63 ± 0.37a	43.11 ± 1.67a	62.93 ± 0.94a	66.83 ± 1.38a	37.53 ± 0.81b	58.51 ± 0.35a	16.52 ± 0.45a
		HSSC	30.38 ± 0.47d	20.83 ± 0.12 cd	9.84 ± 0.26b	38.11 ± 0.35b	51.30 ± 0.39b	49.65 ± 1.08 cd	30.39 ± 1.08c	48.97 ± 1.25c	14.77 ± 0.37 cd
**ANOVA**											
Soil salt-ion concentration (SSC)			**	**	**	**	**	**	**	**	**
Cultivar (C)			ns	**	ns	ns	ns	**	*	**	**
Year (Y)			ns	ns	ns	ns	ns	ns	*	ns	ns
SSC*C			ns	*	ns	ns	ns	ns	*	ns	ns
SSC*Y			*	ns	ns	ns	ns	ns	ns	ns	ns
C*Y			*	ns	ns	ns	ns	ns	ns	ns	ns
SSC*C*Y			ns	ns	ns	ns	ns	ns	ns	ns	ns

LSSC and HSSC represent low soil salt-ion concentration and high soil salt-ion concentration.

Probability levels are performed by ns, * and ** for not significant, 0.05 and 0.01. Different letters within the same column indicate significant difference at p = 0.05 between different treatments during two growing seasons. Data is mean ± SE (n = 3).

### Photosynthetic rate and leaf area index

**Figure 2:**
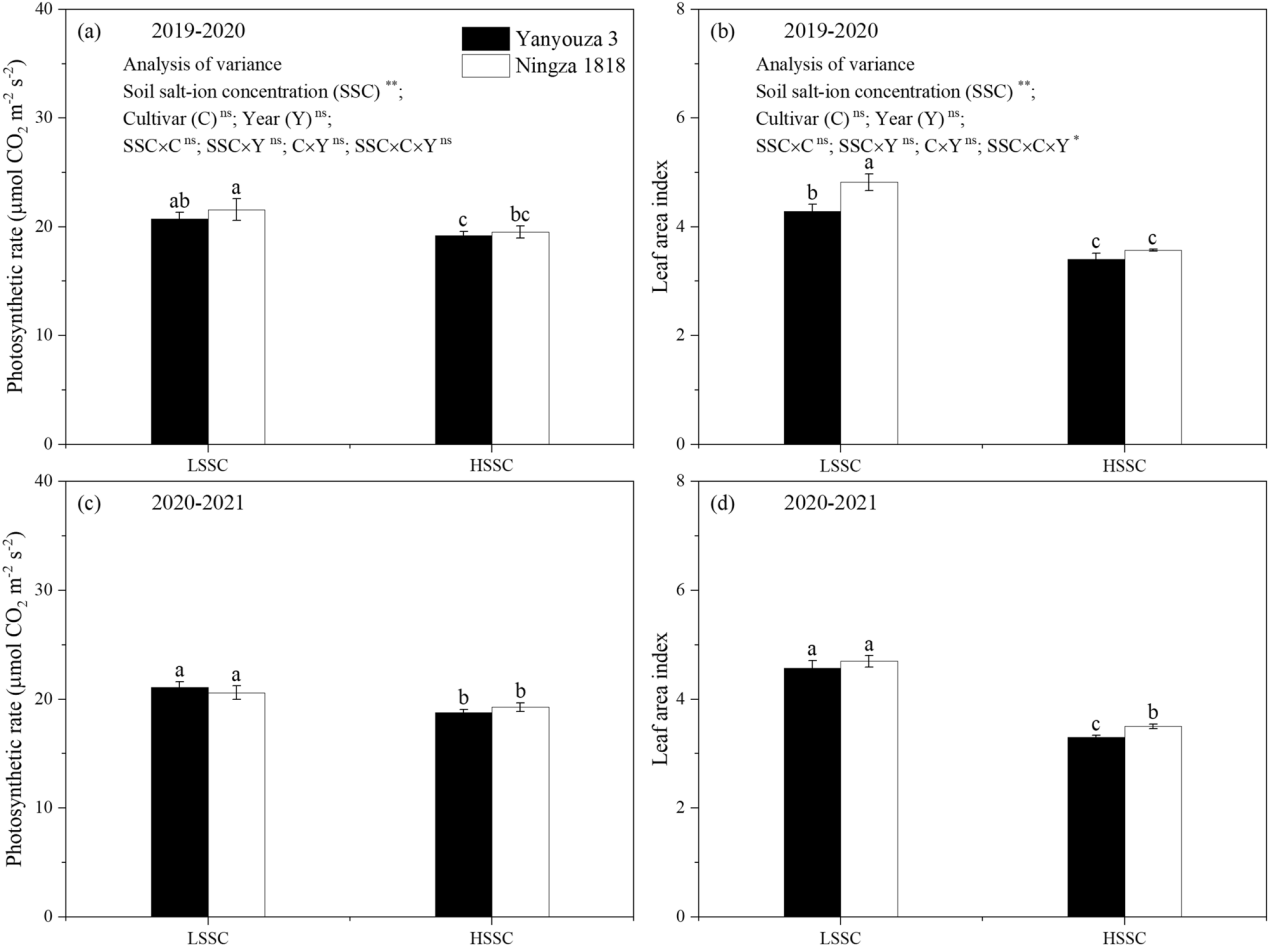
The ANOVA results and photosynthetic rate and leaf area index at early flowering stage under different soil salt-ion concentration. (**a,c**): photosynthesis rate during 2019–2020 and 2020–2021 growing seasons; (**b,d**): leaf area index during 2019–2020 and 2020–2021 growing seasons. LSSC and HSSC represent low and high soil salt-ion concentration. Different letters indicate significant difference at p = 0.05 between different treatments. Data are mean ± SE (n = 3).

The ANOVA results (Fig. 2)showed that soil salt-ion concentration significantly affected photosynthetic rate and leaf area index; cultivar, year and interactions between two factors or three factors showed no significant effect except interactions between three factors on leaf area index.

The variation of photosynthetic rate at early flowering stage between LSSC and HSSC was little but reached a significant level. As compared to LSSC, HSSC averagely decreased the photosynthetic rate at early flowering stage in Yanyouza 3 and Ningza 1818 over two growing seasons by 9.13% and 8.03%, respectively. The leaf area index, ranging from 3.30 to 4.82, was also decreased under HSSC. HSSC treatment decreased leaf area index in Yanyouza 3 and Ningza 1818 by 24.25% and 25.69%, respectively.

## Discussion

In this study, we found that high soil salt-ion concentration decreased the N and C accumulation with more decrement observed in C accumulation than in N accumulation, and inhibited photosynthetic rate and leaf area index, and reduced N translocation efficiency and N utilization efficiency, resulting in reduced biomass accumulation and seed yield.

In our study, the increase in soil salt-ion concentration postponed the time of early flowering stage and maturity stage. Generally speaking, plants exposed to abiotic stress usually accelerate senescence and shorted growth period^26^. Zirgoli et al. found that drought stress can result in the flowering and maturity advancing in canola, and the days to flowering stage and maturity stage under drought stress were decreased by 3.27 and 1.29%, respectively, compared to those under normal condition^27^. In addition, low temperature stress promoted floral initiation, as reported by Luo et al.^28^. However, in the current study, salt stress delayed the early flowering stage and maturity stage and prolong the whole growth period. Meanwhile, salt stress decreased the biomass and N accumulation in canola, with more reduction in biomass accumulation than in N accumulation. Therefore, the plant N content in canola under salt stress was increased. The increased N content in canola may resulted in the prolonged growth period.

N and C are two important elements in plant growth and crop yield formation. Salt stress usually inhibits N and C metabolism in plants. In our study, as compared with LSSC, HSSC significantly decreased the N and C accumulation amount at both early flowering stage and maturity stage. Similar results were reported previously^29^. The decrease in N accumulation may be attributed to the limited activities of enzymes in N metabolism. Previous studies had demonstrated that the activities of nitrate reductase and glutamine synthetase which are the key enzymes in N accumulation are reduced under salt stress^30^. The plant N content in our study was increased due to high soil salt-ion concentration. Additionally, the C/N under HSSC was decreased. These results suggested that C assimilation was more sensitive to salts stress than N assimilation. C and N assimilation together constitute the structure of plants, and the balance of these two physiological processes are vital for plant growth and development. The photosynthesis of plant is a important way to assimilate and accumulate C, and it requires a large amount of N which is closely related to synthesis of photosynthetic enzymes^31,32^. It is generally believed that photosynthesis rate is positively correlated with plant N content. According to Kumar et al., under sulfur optimum application, the leaf photosynthesis rate in canola under enough N supply was 48% higher than that with N-limited treatment^33^. Kuai et al. reported that as N application increasing from 0 to 270 kg N ha^−1^, the leaf photosynthesis rate in canola was increased^34^. Gammelvind et al. also demonstrated that the leaf photosynthesis rate in canola responded linearly to increasing N content in leaf^35^. In current study, although N content increased under salt stress, the photosynthetic rate was decreased. This negative effect on photosynthesis under salt stress may be due to lower activities of photosynthetic enzymes. Generally, photosynthesis depends not only on the amount of these enzymes of photosynthesis but also on the activities of these enzymes^36^. Therefore, the increase in enzyme amount per leaf area due to increasing N content under salt condition was not enough to compensate for the decrease in activities of enzyme, resulting in the decrease in photosynthesis rate. In turn, the N accumulation under salt stress is decreased because of lower energy and carbon skeletons provided by C assimilation^37^. In conclusion, the balance between C and N assimilation of canola plant is destroyed by salt stress, with stronger negative effect on C assimilation. In addition, leaf acts as the main photosynthetic organ of canola at early flowering stage. We found that the leaf area index was also decreased due to HSSC, with more decline than photosynthetic rate, suggesting that the decrease in photosynthetic area is the main reason for the inhibited synthesis of carbohydrate through photosynthesis.

Most of the seed N accumulation in canola are derived from remobilization of N in vegetative organs. A great capacity of N translocation is related to seed yield formation. In our study, the N translocation efficiency in stem and leaf was significantly and positively related with seed N accumulation and seed yield, suggesting that N translocation in vegetative organs is an important source of seed N. However, the N translocation efficiency in all organs decreased with the increase of soil salt-ion concentration, with the most reduction observed in stem. Results agreed with ours were reported that salt stress reduced N translocation in canola^29^, in rice^38^ and barley^39^, suggesting that salt stress prefer to fix N into vegetative organs rather than to transport it into reproductive organs. Ultimately, N utilization efficiency was declined because of lower N translocation efficiency.

## Conclusions

In this field study, HSSC reduced seed yield and postponed the time of early flowering stage and maturity stage. Besides, HSSC decreased N and C accumulation at both growth stages and reduced the C/N, suggesting that salt stress breaks the balance between N and C assimilation and shows stronger negative effects on C assimilation than on N assimilation. Moreover, although the plant N content was increased under salt stress, the photosynthesis rate was reduced. The leaf area index under HSSC was also reduced, with more reduction than photosynthesis rate. In addition, HSSC reduced the N translocation efficiency in all vegetative organs and N utilization efficiency. This means HSSC tend to fix N into vegetative organs rather than transport it into reproductive organs. The findings from this study would help further to understand that salt stress decrease canola seed yield by affecting N and C assimilation and N translocation.

## Materials and methods

### Experimental materials, site and soil conditions

During 2019–2020 and 2020–2021 growing seasons, two hybrid canola cultivars (Yanyouza 3 and Ningza 1818) were planted at the experimental field of Jiangsu Golden Agriculture Shareholding Co., Ltd., Jiangsu, China (33°24′ N, 120°35′ E). The two hybrids were popular winter canola in Middle-Lower Yangtze Area, China. The soil in experiment had a texture of sandy loam. Soils were sampled for the measurement of soil salt-ion concentration, soil organic matter content and soil pH prior to sowing in 2019. The soil chemical properties of the plough layer (0–20 cm) were listed in (Table 9). The soil was sampled and then air-dried at room temperature. Then the soil samples were passed through a 2 mm sieve for measurement (1 soil:5 water). The soil salt-ion concentration in the leachate was determined. Na^+^, K^+^, Ca^2+^ and Mg^2+^ were determined by atomic absorption spectrophotometry. Cl^−^ was determined by silver nitrate titration. HCO_3_^−^ were determined by sulfuric acid titration, SO_4_^2−^ was determined by barium sulfate turbidimetric method. The soil salt-ion concentrations between two treatments were different significantly for some ions, donated by the low soil salt-ion concentration (LSSC) and the high soil salt-ion concentration (HSSC). The difference in the soil salt-ion concentration between LSSC and HSSC was attributed to their difference at altitude of 0.9 and 1.1 m, respectively.

**Table 9:** Soil basic properties in the study.

Treatment	Soil salt-ion concentration (g kg^−1^)								Soil organ matter content (g kg^−1^)	Soil PH
	K^+^	Na^+^	Ca^2+^	Mg^2+^	HCO_3_^−^	Cl^−^	SO_4_^2−^	Total		
LSSC	0.058b	0.488b	0.236b	0.062b	0.383b	0.917b	0.363a	2.507b	16.03a	8.03a
HSSC	0.081a	1.195a	0.301a	0.085a	0.476a	2.171a	0.346a	4.655a	15.68a	8.21a

LSSC and HSSC represent low soil and high soil salt-ion concentration.

Different letters within the same column indicate significant difference at p = 0.05.

### Experimental design

A split plot design was arranged with two soil salt-ion concentrations as main plots and two cultivars as subplots, in three replicates. The plot size was 18 m in length by 2.4 m in width. The canola seeds were manually sown on October 11th in each year and seedling density was adjusted at planting density of 45 × 10^4^ plants ha^−1^ at the fourth-leaf growth stage for all plots (row spacing 0.4 m and plant spacing 0.055 m). Urea (N, 46%), diammonium hydrogen phosphate compound fertilizer (N-P_2_O_5_, 18–46%), potassium sulfate fertilizer (K_2_O, 52%) and boron fertilizer (B, 12%) were applied pre-sowing at rate of 166.0 kg ha^−1^, 326.1 kg ha^−1^, 144.2 kg ha^−1^ and 4.5 kg ha^−1^, respectively, as basal fertilizers. Urea was applied at rate of 293.5 kg ha^−1^ at bolting stage.

### Sampling and measurement

#### Seed yield and biomass accumulation

Ten plants were sampled from each plot at early flowering stage (about 25% of plants begins to blossom). The samples were separated into root, stem and leaf, and dried in an oven for 30 min at 105 °C to deactivate enzymes then again at 80 °C until constant weight to determine the dry weight. Canola was harvested when approximately 90% of pods were yellow. Ten plants were again sampled from each plot. The samples were separated into root, stem, pod and seed. Next, the samples were aired, threshed, dried at 80 °C and weighted. The seed yield was calculated by multiplying the seed yield per plant by density.

Throughout experiment, the deciduous leaves were collected, dried and weighted, at a fixed site (six consecutive rows and in 2 m length each row) from each plot, once every 2 weeks. All the collected deciduous leaves were later separated into pre-anthesis and post-anthesis weights.

#### N accumulation amount, C accumulation amount, N/C and plant N content

The N and C content in different organs was determined using the elemental analyzer (Vario MAX CN, Elementar Co., Germany). The N (C) accumulation amount in specific organ was calculated by multiplying the dry weight by N content in this organ. The C/N was the ratio of C accumulation to N accumulation.

#### N translocation efficiency and N utilization efficiency

The various parameters referring to N translocation efficiency and utilization efficiency of canola in this study were calculated as follows:

- N translocation amount of specific vegetative organ = N accumulation amount of specific organ at early flowering stage−N accumulation amount of specific organ at maturity stage;
- N translocation efficiency of specific vegetative organ = N translocation amount of specific vegetative organ/N accumulation amount of specific vegetative organ at early flowering stage × 100;
- N utilization efficiency = Seed yield/N accumulation amount at maturity (kg kg^−1^).

#### Photosynthetic rate and leaf area index

The photosynthetic rate was measured at early flowering stage using a portable photosynthetic system (LI-COR, Lincoln, NE, USA). The data was obtained from the second and third fully expanding top leaves from 9:00 to 12:00 AM on sunny day. The measurement was performed under a light-saturating photosynthetic photon flux density of 1200 μmol m^−2^ s^−1^. The CO_2_ concentration in the leaf chamber was set at 400 μmol mol^−1^.

Leaf area was measured at early flowering stage using leaf area meter (Model LI-3100, Lincoln, Nebraska). Leaf area index was calculated as the below.

$$\mathrm{Leaf \; area\; index} = \frac{\mathrm{leaf \; area \; per \; plant}\times \mathrm{density}}{\mathrm{planting \; area}}$$

### Statistical analysis

The observation data were compiled with Microsoft Excel 2007, and the analysis of variance (ANOVA) and significance test were conducted using SPSS statistical 20 software (SPSS Inc., Chicago, IL, USA). The mean difference between treatments were separated by Duncan’s multiple range test at significance level of p < 0.05. Graphs were performed using Origin 9.0 software (Origin Lab Corp, Northampton, MA, USA).

### Ethical approval and consent to participate

The seeds were kindly provided by Yangzhou Academy of Agricultural Sciences, Yangzhou, China and Jiangsu Academy of Agricultural Sciences, Jiangsu, China. In this study, the experimental research and field studies on plants, including collection of plant material, complied with relevant institutional, national, and international guidelines and legislation.

## Data Availability

The datasets used and/or analyzed during the current study available from the corresponding author on reasonable request.
